# Double take: A dual-functional Hypr GGDEF synthesizes both cyclic di-GMP and cyclic GMP—AMP to control predation in *Bdellovibrio bacteriovorus*

**DOI:** 10.1371/journal.pgen.1010263

**Published:** 2022-07-21

**Authors:** Aathmaja Anandhi Rangarajan, Christopher M. Waters

**Affiliations:** Department of Microbiology and Molecular Genetics, Michigan State University, East Lansing, Michigan, United States of America; Michigan State University, UNITED STATES

Bacteria possess well-orchestrated signal transduction mechanisms, such as nucleotide second messengers, to tune gene regulation for adaption and survival in variety of environmental conditions [1,2]. Over the last 2 decades, our knowledge of cyclic di-nucleotide (cdNs) second messengers has exploded, revealing that these molecules are key signals that carry out this function in bacteria and eukaryotes [3]. A recent *PLOS Genetics* paper by Lowry and colleagues adds a new twist to cdN signaling, demonstrating for the first time that a cdN synthase can synthesize in vivo 2 cdNs that each have a distinct physiological role [4].

**Fig 1:**
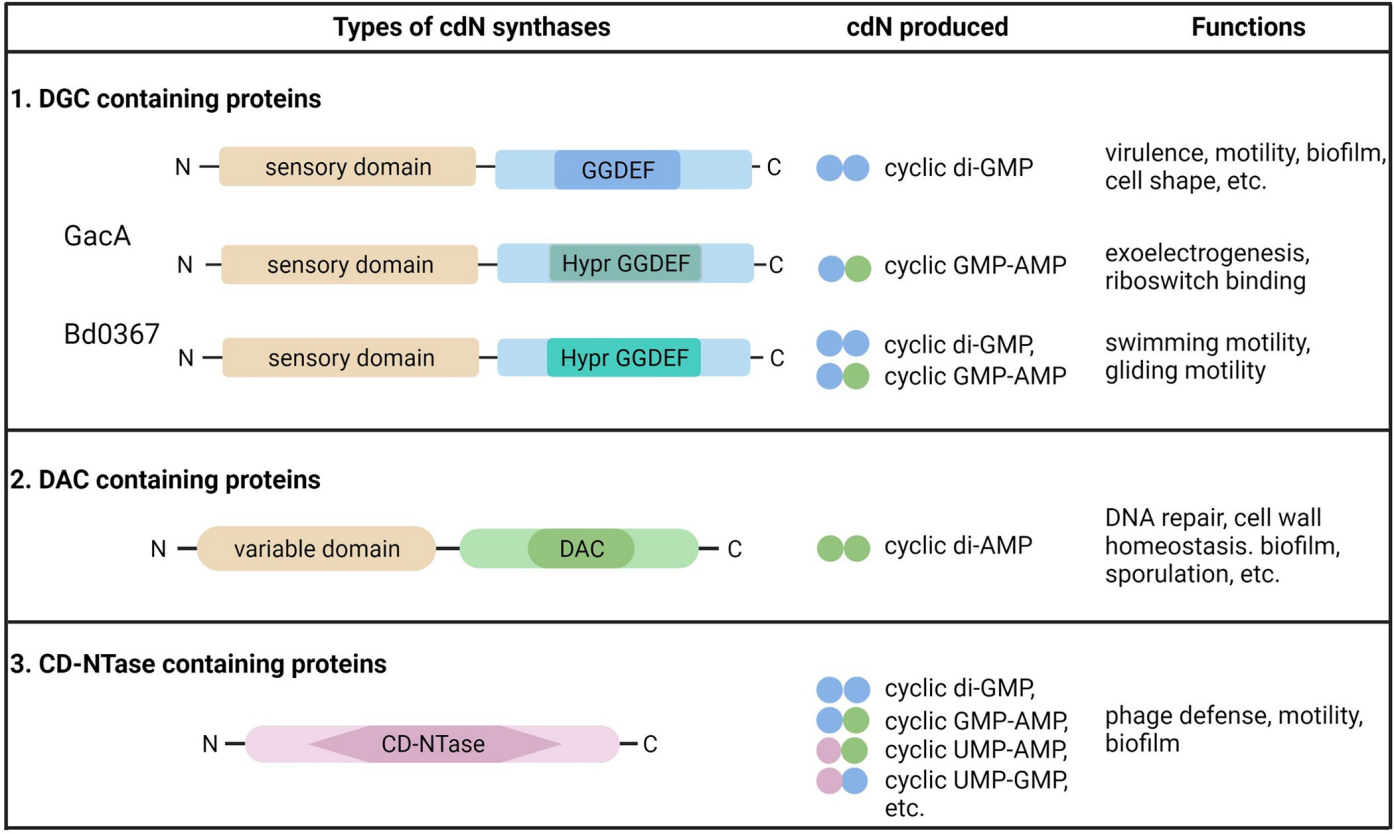
The 3 families of cdN synthases, the cdNs they produce, and their functions. CD-NTase, cGAS/DncV-like nucelotidyl transferase; cdN, cyclic di-nucleotide; DAC, diadenylate cyclase; DGC, diguanylate cyclase.

There are 3 families of cdN synthases that regulate a multitude of biological functions (Fig 1). Cyclic di-GMP (c-di-GMP), the earliest discovered cdN, is synthesized by diguanylate cyclase (DGC) enzymes containing a GGDEF motif [5,6]. C-di-GMP regulates a variety of cellular processes including virulence, motility, biofilm formation, and even cell shape [6–8]. DGCs are generally highly specific for making c-di-GMP, but some DGCs, known as Hybrid promiscuous (Hypr) GGDEFs, which consist of less than 0.2% of all GGDEF domains, synthesize 3′3′ cyclic GMP–AMP (cGAMP) instead of c-di-GMP [9]. Cyclic di-AMP (c-di-AMP) is synthesized by diadenylate cyclase (DAC) domains, and no DACs have been discovered that synthesize other cdNs [10,11]. Cyclic di-AMP plays a crucial role in DNA repair, cell wall homeostasis, osmotic homeostasis, sporulation, and biofilm formation in predominantly gram-positive bacteria [12,13]. Another broadly classified group of enzymes, the cGAS/DncV-like nucelotidyl transferase (CD-NTase), which are also widely present in several bacterial phyla, produces a variety of purine, pyrimidine, and purine-pyrimidine hybrid cdNs and even cyclic tri-nucleotides [14]. This family of enzymes includes DncV in *Vibrio cholerae* and cGAS in metazoans, and it is the most promiscuous of the cdN synthase family [14,15]. cGAMP synthesized by CD-NTases mediates bacterial phage defense through an abortive infection mechanism, while cGAMP synthesized by Hypr GGDEFs controls exoelectrogenesis in *Geobacter sulfurreducens* [15–21].

**Fig 2:**
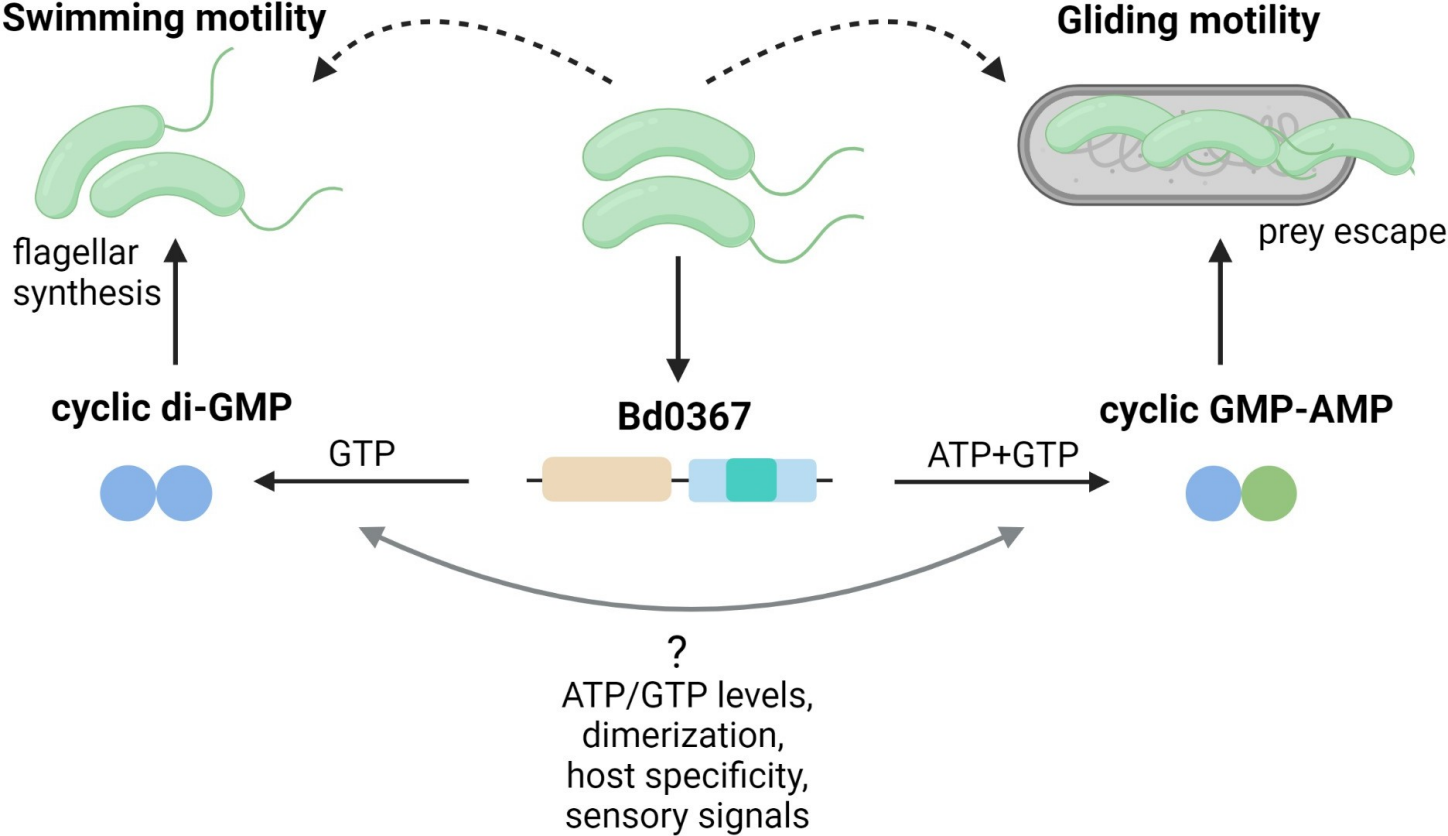
The dual role of Bd0367 in *B*. *bacteriovorus*. Bd0367 can produce cGAMP and c-di-GMP, which control gliding motility and swimming motility, respectively. The signals that regulate synthesis of c-di-GMP or cGAMP by Bd0367 are unknown.

Lowry and colleagues have expanded our understanding of these 3 cdN synthase enzyme families by discovering a novel Hypr GGDEF protein in *Bdellovibrio bacteriovorus*, Bd0367, which synthesizes both cGAMP and c-di-GMP to regulate gliding and swimming motility, respectively (Fig 2) [4]. *B*. *bacteriovorus* is gram-negative bacterium that preys upon other bacteria by invading their periplasm and stealing the host cell’s nutrients for growth before ultimately escaping the exhausted prey using gliding motility [22,23]. While a Δ*bd0367* mutant of *B*. *bacteriovorus* could enter prey cells, it was unable to glide and escape from the prey to complete the predatory life cycle, and this defect was previously attributed to a loss of c-di-GMP synthesis. [24]. However, Bd0367 is actually a Hypr GGDEF because it encodes a key amino-terminal serine (S124) in its GGDEF domain that allows utilization of GTP and ATP while standard GGDEFs encode an aspartate at this position [18]. Whereas the WT Bd0367 enzyme primarily synthesizes 3′3′-cGAMP in vitro, generation of the Bd0367^S124D^ mutant enzyme rendered it solely able to synthesize c-di-GMP. Moreover, complementation of the Δ*bd0367* mutant with the *bd0367*^S124D^ allele abolished in vivo cGAMP synthesis and did not restore functional gliding motility or escape from prey cells [18]. This is an exciting finding as it expands the realm of bacterial cGAMP signaling into *Bdellovibrio*. For reasons that are not yet understood, Δ*bd0367* mutants only grow outside of the host if they acquire a null suppressor mutation in the flagellar chaperone *fliS*. However, the *bd0367*^S124D^ mutant does not mimic the Δ*bd0367* mutant as it can grow outside of the host without the need for additional suppressor mutations in *fliS*, suggesting c-di-GMP synthesis by Bd0367 has a functional role in the cell that abrogates the need for *fliS* suppressor mutations during host independent growth. This is the first in vivo example showing a single Hypr GGDEF containing enzyme produces in vivo 2 different cdNs, 3′3′-cGAMP and c-di-GMP, in which each regulate a distinct physiological process.

The predatory bacterium *B*. *bacteriovorus* undergoes a complex life cycle involving several sequential events: hunt and attachment to prey, prey entry, intracellular growth forming a bdelloplast, replication inside the bdelloplast, rupture, and exit of the prey cell [22,23]. The physiological conditions and the growth phase that triggers Bd0367 enzymes to produce cGAMP or c-di-GMP is unknown, but it could depend on several factors including the specific host, ATP/GTP concentrations in that host, dimerization of the protein, or unidentified signals affecting the amino-terminal receiver domain. *B*. *bacteriovorus* has only 4 GGDEF containing proteins, whereas other bacteria like *Vibrios*, *Escherichia coli*, or *G*. *sulfurreducens* have dozens of DGCs [23–26]. Due to the small number of GGDEF proteins present in *B*. *bacteriovorus*, Bd0367 may have evolved to produce more than 1 cdN to increase signaling flexibility, allowing it to respond to specific signals to successfully complete its predatory lifecycle.

The well-characterized Hypr GGDEF protein GacA in *G*. *sulfurreducens* synthesizes c-di-GMP, cGAMP, and c-di-AMP in vitro but only an in vivo role for cGAMP has been determined [18]. Likewise, CD-NTases from many bacteria produce more than 1 dinucleotide signal in vitro, but their in vivo activity is less characterized [14]. Hypr GGDEF containing proteins are present across different genera [9]. It is possible that these Hypr GGDEF and other CD-NTases produce more than 1 functional cdN in vivo under specific physiological conditions. This might be especially true for predatory bacteria like *Bdellovibrio* or species that encode fewer GGDEF or Hypr GGDEF containing proteins, allowing an expansion of the cdN repertoire with fewer synthases. The research of Lowry and colleagues raises the possibility that cdN synthases could produce multiple signals, each with a distinct regulatory role, and it will be intriguing to discover which synthases have this capability [4].
